# Editorial: Peptide/Polyketide Molecules From Marine Macro and/or Microorganisms

**DOI:** 10.3389/fchem.2020.00490

**Published:** 2020-07-14

**Authors:** Valeria Costantino, Marie-Lise Bourguet-Kondracki, Efstathia Ioannou

**Affiliations:** ^1^The Blue Chemistry Lab, Dipartimento di Farmacia, Università degli Studi di Naples Federico II, Naples, Italy; ^2^Laboratoire Molécules de Communication et Adaptation des Micro-organismes, UMR 7245 MNHN-CNRS, Muséum National d'Histoire Naturelle, Paris, France; ^3^Section of Pharmacognosy and Chemistry of Natural Products, Department of Pharmacy, School of Health Sciences, National and Kapodistrian University of Athens, Athens, Greece

**Keywords:** genome mining, non-ribosomal peptides, molecular networking, metabolomics, natural products, peptide/polyketide molecules

This special issue was intended to be a collection of studies focused on the ability of marine macroorganisms and microorganisms to assemble complex biosynthetic machineries for the production of a huge variety of natural products.

Polyketides are a class of metabolites composed of simple C_2_-C_4_ building blocks (acetyl, propionyl, or butyryl), assembled from their respective activated forms malonyl-CoA, methylmalonyl-CoA, and ethylmalonyl-CoA through the polyketide synthase (PKS) pathway. Peptides can be either ribosomally synthesized and posttranslationally modified (RIPPs) or assembled through mega-synthetases referred to as non-ribosomal peptide synthetases (NRPS) in an RNA-independent synthetic pathway. Interestingly, NRPS modules can easily work with PKS modules, resulting in a different biosynthetic pathway which produces a number of novel polyketide–peptide metabolites. These molecules are, without any doubt, the new frontiers for identifying novel lead compounds in drug discovery.

Studies in this field are strongly facilitated by the powerful technologies set up in the last 15 years that combine molecular biology with chemical and bioinformatic expertise.

Papers collected in this special issue highlight this concept, as well as the need for an interdisciplinary approach for advanced and effective studies in the field of natural products. In addition, the genome mining approach has revealed an unprecedented biosynthetic potential for the discovery of novel natural products that have attracted the attention of many groups of researchers all over the world.

“Genome mining” technology allows the identification of biosynthetic gene clusters never reported before within the genome of sequenced organisms. Gene clusters encode proteins to be involved in the biosynthesis of natural products that, in many cases, have never been elucidated before by natural product researchers. These secondary metabolites, having unique structures, are produced by pathways, the so-called cryptic biosynthetic pathways, previously undescribed. As a result, this technology reveals an exponentially increasing amount of DNA sequence data.

The potential of genome mining has been evidenced in the work reported in this special issue by Della Sala et al. that succeeded in the identification of the putative NRPS gene cluster responsible for the biosynthesis of the bioactive cyclohexapeptide thermoactinoamide A, produced by *Thermoactinomyces vulgaris* DSM 43016.

It is worth noting that in this paper, genome mining has been combined with a powerful bioinformatic tool, Molecular Networking (GNPS, gnps.ucsd.edu), a web-based open-access system that allows fast dereplication of complex metabolome based on tandem mass spectrometry data (MS/MS).

Integrating genome mining with LC-HRMS/MS molecular networking-based investigation of the microbial metabolome, 10 analogs of thermoactinoamide A were identified, with five of them—thermoactinoamides G–K—being new compounds.

The “overexpression” technology is the classical genetic approach for exploring biological pathways and activating silent biosynthetic pathways that, as a consequence, produce chemical diversity in secondary metabolism. An interesting contribution to this field came from Wang et al. who described the overexpression of the PbrlaeA gene of the fungus *Penicillium brocae* HDN-12-143 and the resulting isolation of four compounds, including fumigatin chlorohydrin and the new compound iso-fumigatin chlorohydrin.

Besides overexpression, the expression of a characterized gene cluster in a well-studied heterologous host has emerged as a powerful technology. Applied by Rodríguez Estévez et al. to the discovery of the biosynthetic genes for the production of the antibiotic nybomycin (*nyb* genes) in *Streptomyces albus* ssp. *chlorinus*, it led to the identification of a novel metabolite named benzanthric acid, in addition to the isolation of nybomycin.

Chemical epigenetic manipulation has been proven a fruitful pathway for producing new types of specialized metabolites, as illustrated in the exciting study of Wu et al. on the fungal strain *Cochliobolus lunatus* TA26-46, when cultivated with a DNA methyltransferase inhibitor.

Peptidic synthesis was also exploited in order to identify the smallest fragments of a marine neuroprotective toxin isolated from *Anemonia sulcata*, named blood depressing substance-I (BDS-I). This was revealed to be able to block the Kv3.4 channel, which is recognized as a possible target in Alzheimer's disease (AD). The novel peptide, identified by Ciccone et al. as the smallest active BDS-I amino acid sequence, was named BDS-I. It was shown to exert a Kv3.4 inhibitory activity, preventing Aβ peptide-induced cell death. This study provides a novel approach in the development of potential neuroprotective drugs, especially for the treatment of AD.

A South Korean–Vietnamese collaboration (Thuan et al.) provided a comprehensive update review on origins, structures, biological activities, and mechanism of actions of major compounds from selected Cyanobacteria genera (*Nostoc, Lyngbya*, and *Microcystis*). Macrocyclic depsipeptides named cryptophycins as well as hybrids PKS/NRPS curacins have emerged as potential anticancer compounds in preclinical and clinical trials. The cyclic depsipeptides micropeptins and the linear peptides aeruginosins have been highlighted as significant protease inhibitors. New trends, including multi-omic approaches and biotechnological production, have been unveiled for enhanced production of target compounds.

We really think that research groups have been “motivated” to move from the traditional way of analyzing metabolomic content of natural extracts based on bioassay-guided separation and structural elucidation to the innovative “genome mining” approach yielding exciting data worthy of dissemination to academic and public fora. It is becoming more apparent that an intersectoral approach to the study of nature, generally speaking, is needed, and young researchers are urged to share this point of view and be ready to work together, combining different expertise and experience. Are we ready for that?

## Author Contributions

VC conceived the editorial. VC, M-LB-K, and EI equally contributed to the writing. All authors contributed to the article and approved the submitted version.

## Conflict of Interest

The authors declare that the research was conducted in the absence of any commercial or financial relationships that could be construed as a potential conflict of interest.

